# Discordance Between Anatomical Nerve Root Compression and Functional Radiculopathy in Patients with Lumbosacral Transitional Vertebrae

**DOI:** 10.3390/jcm15103809

**Published:** 2026-05-15

**Authors:** Yunjin Nam, Jae Hyuk Yang, Dong-Gune Chang, Seung Woo Suh

**Affiliations:** 1 Department of Orthopedic Surgery, Korea University Guro Hospital, Seoul 08308, Republic of Korea; nam.yunjin@gmail.com; 2 Department of Orthopedic Surgery, Korea University Anam Hospital, Seoul 02841, Republic of Korea; kuspine@naver.com; 3 Department of Orthopedic Surgery, Inje University Sanggye Paik Hospital, Seoul 01757, Republic of Korea; dgchangmd@gmail.com

**Keywords:** lumbosacral transitional vertebrae, radiculopathy, electromyography, magnetic resonance imaging, nerve root compression, anatomical –functional discordance

## Abstract

**Background/Objectives**: Lumbosacral transitional vertebrae (LSTV) are associated with alterations in spinal anatomy and biomechanics that may complicate localization of symptomatic nerve root pathology. However, the relationship between anatomically defined nerve root compression on magnetic resonance imaging (MRI) and electrophysiologically identified radiculopathy on electromyography (EMG) remains insufficiently characterized. This study aimed to evaluate concordance between MRI-identified nerve root compression and EMG-identified radiculopathy and to investigate whether transitional morphology influences this relationship. **Methods**: A retrospective analysis was performed in patients with LSTV who underwent both MRI and needle EMG for single-level lumbosacral radiculopathy. Transitional vertebrae were classified as lumbarization or sacralization and further categorized as complete or partial. Concordance between the anatomical level of nerve root compression and the electrophysiological level of radiculopathy was assessed. Clinical and radiographic variables potentially associated with discordance were analyzed. **Results**: Twenty-nine patients were included. Concordance between MRI-defined nerve root compression and EMG-identified radiculopathy was observed in 27.6% of cases, whereas 72.4% demonstrated discordance. The degree of transitional morphology significantly influenced concordance patterns, with complete transitional vertebrae showing a higher rate of discordance compared with partial transitions. **Conclusions**: In this exploratory cohort, patients with LSTV showed a high rate of anatomical–functional discordance between MRI and EMG findings. Complete transitional morphology is associated with increased discordance, suggesting altered functional expression of nerve root pathology. These findings suggest that anatomical and electrophysiological assessments may provide complementary information during diagnostic evaluation in selected patients with LSTV.

## 1. Introduction

Lumbosacral transitional vertebrae (LSTV) represent one of the most common congenital variations in the spine and are characterized by morphological alterations at the lumbosacral junction, including sacralization of the lowest lumbar vertebra and lumbarization of the uppermost sacral segment [1]. These anatomical variations may modify segmental alignment, load transmission, and neural exit orientation, thereby potentially influencing both the structural and functional characteristics of lumbosacral nerve roots [2,3,4]. The reported prevalence of LSTV ranges from approximately 4% to 30% in the general population, reflecting heterogeneity in diagnostic criteria and morphological classification [5,6].

Clinically, LSTV has long been recognized as a source of diagnostic uncertainty in vertebral numbering and surgical level identification [7]. Inaccurate level identification in the presence of transitional anatomy may increase the risk of wrong-level surgery and misinterpretation of imaging findings [8]. Beyond numbering challenges, emerging evidence suggests that transitional morphology may influence the functional expression of radiculopathy, raising the possibility that the anatomically compressed nerve root does not necessarily correspond to the clinically symptomatic level [9]. This discrepancy may represent a clinically important diagnostic pitfall, particularly when clinical decision-making relies predominantly on structural imaging.

Recent studies have emphasized that lumbosacral biomechanical alterations and segmental instability may be associated with pain, functional impairment, and disability, highlighting the importance of considering both structural and functional factors in spinal disorders [10,11]. These findings suggest the need for integrated diagnostic evaluation rather than reliance on anatomical imaging alone. In patients with LSTV, this concept may be particularly relevant, as transitional vertebral morphology itself may alter the functional characteristics of lumbosacral nerve roots. For example, Chang et al. reported that in patients with S1 lumbarization, the S1 nerve root exhibited functional features resembling those of the L5 root [12]. Similarly, Hinterdorfer et al. demonstrated that lumbarization may result in functional redistribution of sacral nerve root expression, with the S2 root showing characteristics analogous to the S1 root in individuals with a typical five-lumbar-segment spine [13]. These observations suggest that morphological variation at the lumbosacral junction may be associated with alterations in the functional characteristics and segmental expression of lumbosacral nerve roots [14].

Magnetic resonance imaging (MRI) is the primary modality for identifying structural nerve root compression, whereas electromyography (EMG) provides complementary information regarding the functional status of nerve roots [15,16]. Although EMG is widely used to localize radiculopathy and evaluate functional nerve root involvement, its findings should be interpreted in conjunction with clinical presentation and imaging studies, as false-negative results and technical limitations may occur depending on disease chronicity, examiner expertise, and patient-specific factors [17]. However, the relationship between imaging-defined compression and electrophysiologically identified radiculopathy remains incompletely understood in patients with transitional vertebrae. Most previous studies have focused primarily on the radiological classification, vertebral numbering, or biomechanical consequences of transitional anatomy, particularly in relation to wrong-level surgery and adjacent segment degeneration [1,9,18]. In daily clinical practice, however, surgical decision-making is often complicated by discrepancies between imaging findings and patient symptoms, especially in patients with LSTV [19]. Despite the clinical importance of identifying the clinically relevant symptomatic nerve root level, the functional correspondence between imaging-defined nerve root compression and electrophysiologically identified radiculopathy has remained insufficiently explored [20,21]. In particular, whether transitional morphology influences this anatomical–functional concordance has not been systematically evaluated [12,13,14].

Against this background, the present study aimed to evaluate the concordance between MRI-identified nerve root compression and EMG-identified radiculopathy in patients with LSTV. By focusing on potential discrepancies between anatomical and functional findings, this study aimed to clarify diagnostic discordance in this clinically relevant condition.

## 2. Materials and Methods

This study was conducted as a single-centre retrospective observational study. Institutional Review Board approval was obtained from our institution, and the requirement for informed consent was waived owing to the retrospective design of the study. Consecutive patients who visited our hospital between 2005 and 2024 were screened for eligibility. Inclusion criteria were as follows: (1) presence of lumbosacral transitional vertebrae identified based on vertebral numbering using whole-spine sagittal MRI, (2) single-level nerve root compression identified on lumbar MRI, including disc herniation, lateral recess stenosis, or foraminal stenosis, and (3) single-level radiculopathy identified by electrophysiological findings on EMG and correlated with clinical symptoms. Patients with multilevel pathology or central canal stenosis with potential cauda equina involvement were excluded to minimize confounding effects of multiple nerve root involvement.

Vertebral numbering was performed on whole-spine sagittal MRI by sequentially counting vertebral segments from the cranial end of the spine, assuming the presence of 7 cervical and 12 thoracic vertebrae [22,23]. Segment numbering was based on the standard anatomical convention of five lumbar vertebrae and five sacral vertebrae, regardless of transitional morphology. Accordingly, in cases of lumbarization, the transitional segment was designated as S1 despite exhibiting lumbar-type morphology, whereas in cases of sacralization, the transitional segment was designated as L5 despite partial or complete fusion with the sacrum. This standardized numbering approach was applied consistently across all patients.

After vertebral numbering, LSTV was defined based on morphological characteristics at the lumbosacral junction on lumbar MRI [1,9]. Transitional anatomy included either sacralization of the lowest lumbar vertebra or lumbarization of the uppermost sacral segment. Transitional vertebrae were further classified as complete or partial according to previously described morphological criteria [24,25]. Complete lumbarization was defined as complete morphological separation of the uppermost sacral segment from the sacral mass, resulting in a vertebral segment demonstrating lumbar-type structural characteristics. Partial lumbarization was defined as incomplete separation of the uppermost sacral segment with residual structural continuity to the sacrum [24]. Complete sacralization was defined as complete osseous fusion of the lowest lumbar vertebra to the sacrum, either unilaterally or bilaterally. Partial sacralization was defined as incomplete fusion characterized by pseudoarticulation or partial bony continuity between the transverse process of the lowest lumbar vertebra and the sacral ala [25].

Nerve root compression was determined based on formal MRI reports interpreted by board-certified radiologists and reviewed in conjunction with clinical findings by the spine surgeon. In cases of disagreement between the radiologist and spine surgeon, the radiologist’s interpretation was adopted as the final determination. The corresponding root level was determined according to vertebral numbering sequentially established from whole-spine sagittal MRI to minimize segmental misidentification, as previously recommended in patients with transitional vertebrae [22].

Needle EMG studies were performed on the lumbosacral paravertebral muscles and selected lower extremity muscles, including the vastus lateralis, tibialis anterior, extensor hallucis longus, peroneus longus, flexor digitorum longus, tensor fasciae latae, gluteus maximus, and the medial head of the gastrocnemius [16]. All EMG findings were reviewed and interpreted by board-certified rehabilitation medicine specialists. Electrophysiological evaluation included assessment of insertional activity, abnormal spontaneous activity, motor unit action potentials, and recruitment patterns [15]. The level of radiculopathy on EMG was determined based on the pattern of muscle involvement using established electrodiagnostic principles [26,27]. Muscles were evaluated according to their predominant segmental innervation. In general, tibialis anterior was primarily associated with either L4 or L5 involvement, extensor hallucis longus with L5 involvement, and gastrocnemius with S1 involvement. Electrophysiological abnormalities were considered significant when identified in at least two muscles corresponding to a single myotomal distribution [16]. When findings involved multiple muscles, the level was determined based on the dominant pattern of involvement. Radiculopathy identified on EMG was interpreted as a functional correlate of clinical radiculopathy rather than a definitive diagnostic gold standard.

Patients were categorized into two groups according to the concordance between MRI findings and EMG results. The concordant group included patients in whom the level of nerve root compression identified on MRI corresponded to the level of radiculopathy identified by electrophysiological findings on EMG. The discordant group included patients in whom the level of anatomical compression did not correspond to the electrophysiologically identified radiculopathy. For example, if MRI demonstrated S1 nerve root compression whereas EMG identified L5 radiculopathy, the case was classified as discordant.

The level of the iliac crest was evaluated on lumbar spine plain radiographs. In patients with a typical lumbosacral configuration, the iliac crest located at the L4–5 disc space was defined as normal [28]. Levels above the L4 vertebral body were classified as high, whereas levels below the L5 vertebral body were classified as low. In the presence of S1 lumbarization, the reference level was adjusted to the L5–S1 disc space, while in cases of L5 sacralization, the L3–4 disc space was used as the reference to account for segmental variation associated with transitional anatomy.

The level of the conus medullaris was determined on MRI. Based on the commonly reported anatomical range of conus termination between L1 and L2 in adults, termination at the L1–2 disc space was operationally defined as normal for categorical analysis [29]. Termination above the L1 vertebral body was classified as high, whereas termination below the L2 vertebral body was classified as low. In the presence of S1 lumbarization, the reference level was adjusted to the L2–3 disc space, while in cases of L5 sacralization, the T12–L1 disc space was used as the reference.

Clinical presentation at the time of diagnosis was also recorded, including pain and motor deficits such as ankle dorsiflexion weakness, great toe extension weakness, and ankle plantar flexion weakness. For comparative analysis, patients were further categorized as having pain only or motor deficit present.

Demographic data, including sex and age, were also recorded. Normality of continuous variables was assessed before selecting parametric or non-parametric tests. For the comparison of continuous variables between groups, Student’s t-test or Mann–Whitney U test was used, while categorical variables were compared using Pearson chi-square test or Fisher’s exact test. In addition, a post hoc power analysis was performed for the comparison of discrepancy rates between complete and partial LSTV groups, which represented the primary subgroup comparison of the study. All statistical tests were performed with a significance level set at *p* < 0.05. The statistical analyses were conducted using SPSS (version 27.0.0; IBM Corp., Armonk, NY, USA).

## 3. Results

A total of 29 patients with LSTV and single-level radiculopathy identified by electrophysiological findings on EMG were included. Baseline demographic and anatomical characteristics are summarized in Table 1. The mean age was 57.9 ± 12.6 years, and disc herniation was the most common underlying pathology. S1 lumbarization was observed in 19 patients, whereas L5 sacralization was present in 10 patients. MRI most frequently demonstrated S1 nerve root compression, whereas EMG findings predominantly indicated L5 radiculopathy. Regarding presenting symptoms, pain only was observed in 18 patients, while 11 patients presented with pain accompanied by motor weakness, including ankle dorsiflexion weakness, great toe extension weakness, ankle plantar flexion weakness, or combined deficits. Discordance between anatomical compression and electrophysiological localization was observed in the study population.

The distribution of EMG-identified radiculopathy levels and the associated patterns of dominant muscle involvement according to LSTV type are summarized in Table 2. In both lumbarization and sacralization groups, L5 radiculopathy was characterized predominantly by involvement of the peroneus longus and tibialis anterior muscles. S1 radiculopathy was more frequently associated with gastrocnemius and gluteus maximus involvement.

**Figure 1:**
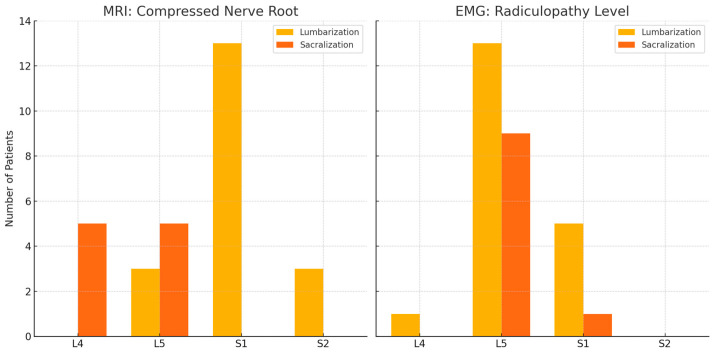
Bar graphs illustrate the distribution of patients by lumbosacral transitional vertebrae (LSTV) type, including lumbarization (yellow, *n* = 19) and sacralization (orange, *n* = 10). The left panel shows the compressed nerve root level identified on magnetic resonance imaging (MRI), while the right panel presents the level of radiculopathy identified by electrophysiological findings on electromyography (EMG). In the lumbarization group, S1 root compression was most frequently observed on MRI, whereas L5 radiculopathy was the predominant finding on EMG. In the sacralization group, both L4 and L5 root compressions were identified on MRI, with L5 radiculopathy most observed on EMG. These findings suggest discordance between anatomical nerve root compression and functional radiculopathy patterns in patients with LSTV.

When stratified by LSTV type, distinct differences in the distribution of compressed nerve roots and radiculopathy levels were observed (Figure 1). In the lumbarization group (*n* = 19), MRI most demonstrated S1 root compression (*n* = 13), whereas EMG most frequently identified L5 radiculopathy (*n* = 13). In the sacralization group (*n* = 10), MRI demonstrated L4 and L5 root compression in 5 patients each. Corresponding EMG findings showed L5 radiculopathy in 9 patients and S1 radiculopathy in 1 patient.

Overall, concordance between MRI-identified nerve root compression and EMG-identified radiculopathy was observed in 8 patients (27.6%), while 21 patients (72.4%) demonstrated discordance. Among patients with S1 lumbarization, 13 exhibited S1 root compression on MRI, of whom 11 showed L5 radiculopathy on EMG. Among these patients, all five patients with L4 root compression in the sacralization group demonstrated L5 radiculopathy on EMG.

No significant differences in concordance were observed according to age, sex, motor weakness, LSTV type, conus medullaris level, iliac crest height, or rib number. However, the degree of transitional morphology was significantly associated with discordance. Patients with complete transitional vertebrae showed a significantly higher rate of discordance compared to those with partial transitions (93.8% vs. 46.2%, respectively, p = 0.010) (Table 3). The estimated post hoc statistical power for this subgroup comparison was 86.4% at a two-sided alpha level of 0.05.

**Figure 2:**
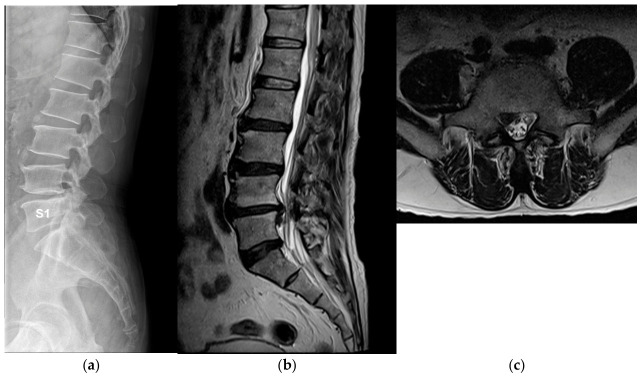
Representative discordant case in a patient with complete lumbarization. A 46-year-old male patient demonstrated complete lumbarization of S1. (**a**) Lumbar spine radiographs show complete separation of the uppermost sacral segment from the sacral mass, consistent with complete lumbarization. (**b**) Sagittal lumbar MRI demonstrates inferiorly migrated disc herniation at the anatomically defined L5–S1 level. (**c**) Axial MRI shows right paracentral disc herniation causing compression of the S1 nerve root. Clinically, the patient presented with ankle dorsiflexion and great toe extension weakness. Electrophysiological evaluation demonstrated abnormal findings in the tibialis anterior, peroneus longus, and tensor fasciae latae muscles, consistent with L5 radiculopathy. These findings illustrate discordance between MRI-defined anatomical compression and EMG-identified functional nerve root involvement.

**Figure 3:**
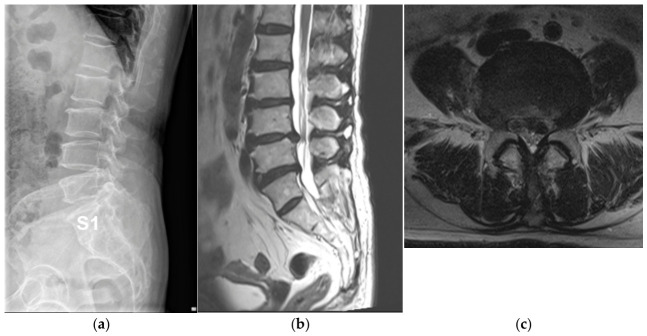
Representative concordant case in a patient with partial lumbarization. A 62-year-old female patient demonstrated partial lumbarization of S1. (**a**) Lumbar spine radiographs show transitional anatomy consistent with partial lumbarization. (**b**) Sagittal lumbar MRI demonstrates disc herniation at the L4–5 level. (**c**) Axial MRI shows left L5 nerve root compression. Clinically, the patient presented with pain without motor weakness. Electrophysiological evaluation demonstrated abnormal findings in the tibialis anterior and peroneus longus muscles, consistent with L5 radiculopathy. These findings demonstrate concordance between MRI-defined anatomical compression and EMG-identified functional nerve root involvement.

Illustrative cases from the concordant and discordant groups are shown in Figure 2 and Figure 3.

## 4. Discussion

The present study showed a high rate of discordance between anatomical nerve root compression identified on MRI and functional radiculopathy identified by electrophysiological findings on EMG in patients with LSTV. Only 27.6% of patients showed concordance between anatomical and electrophysiological findings, while 72.4% exhibited mismatch. These results suggest that in the presence of transitional vertebral anatomy, conventional assumptions regarding the relationship between structural pathology and functional nerve root involvement may not be reliable. Rather than reflecting simple diagnostic inaccuracy, this discordance may indicate variation in the functional expression of nerve root pathology associated with transitional morphology.

Importantly, the present findings indicate that the anatomical labelling of vertebral segments does not necessarily correspond to the physiological identity of the affected nerve root in LSTV patients. This observation may have implications for diagnostic reasoning and therapeutic decision-making. The concept that transitional vertebrae may alter the functional organization of nerve roots challenges traditional segment-based interpretation of imaging findings and underscores the need for integrative diagnostic approaches that incorporate both structural and functional assessments [12].

Another key finding of this study is that the degree of transitional morphology was significantly associated with concordance patterns. Patients with complete transitional vertebrae showed a significantly higher rate of discordance compared with those with partial transitions. In such cases, nerve root function may follow morphological rather than conventional anatomical numbering, leading to increased discordance between imaging-defined compression and electrophysiological findings, whereas partial transitional morphology may preserve relatively greater anatomical–functional concordance.

Several mechanisms may explain the observed mismatch between anatomical compression and functional radiculopathy in LSTV patients. Transitional vertebral morphology may alter the spatial orientation and course of nerve roots, leading to variations in neural tension, mobility, and mechanical stress distribution [22]. Additionally, changes in segmental biomechanics associated with transitional anatomy may influence patterns of degenerative change, potentially shifting the functional burden to adjacent or morphologically analogous neural structures [3]. Another important consideration is that neural function may be more closely linked to vertebral morphology than to strict anatomical numbering [22]. In cases of lumbarization, the morphological configuration of the transitional segment may create a functional environment resembling that of a typical lumbar vertebra, resulting in nerve root behaviour that aligns more closely with morphological identity rather than conventional segmental labelling [30]. This mechanism may be particularly relevant in cases of complete transitional morphology, where structural transformation is more pronounced and may contribute to greater anatomical –functional discordance. These findings suggest that radiculopathy in LSTV patients should not be conceptualized solely as localized anatomical compression, but rather as the combined effect of morphological configuration, biomechanical adaptation, and neural functional variation [9,31].

In general, EMG interpretation of lumbosacral radiculopathy is based on established patterns of segmental muscle involvement [26,27]. L4 radiculopathy is typically associated with abnormalities in the quadriceps and tibialis anterior muscles, L5 radiculopathy commonly involves the tibialis anterior, extensor hallucis longus, peroneus longus, and tensor fasciae latae muscles, whereas S1 radiculopathy frequently affects the gastrocnemius and gluteus maximus muscles. In the present study, the dominant muscle involvement patterns identified on EMG were generally consistent with these established electrophysiological distributions. However, despite these typical EMG patterns, the electrophysiologically identified radiculopathy levels frequently did not correspond to the anatomically compressed nerve root levels identified on MRI. These findings suggest that the observed discordance is unlikely to reflect arbitrary EMG interpretation, but rather may indicate altered functional expression of nerve root pathology associated with transitional anatomy.

Complete transitional vertebrae may be associated with greater variation in lumbosacral plexus organization during developmental segmentation. Because complete lumbarization or sacralization involves more profound structural redefinition of the lumbosacral junction, the functional identity of the corresponding nerve roots may not strictly correspond to conventional vertebral numbering [12,30]. This may result in variation in neural segmental distribution and altered functional expression beyond simple morphological change. Such developmental variation may partly explain why patients with complete transitional morphology showed significantly greater anatomical–functional discordance than those with partial transitions in the present study. Although the sample size was modest, this finding suggests that the degree of transitional anatomy may meaningfully influence anatomical–functional mismatch. Complete LSTV may involve more extensive structural reorganization of the lumbosacral junction, which could contribute to greater discordance between structural compression and functional radiculopathy. This finding should be interpreted cautiously, but it highlights the need for particular clinical attention in patients with complete transitional morphology.

Previous studies have reported that LSTV can influence spinal biomechanics, load distribution, and the development of degenerative pathology at adjacent levels [3,32,33]. In particular, lumbarization of S1 has been reported to produce functional characteristics resembling those of the L5 nerve root [12]. Similarly, anatomical and electrophysiological studies have suggested that morphological resemblance between a lumbarized S1 segment and a typical L5 vertebra may lead to altered neural function patterns and atypical symptom localization [13,14]. However, previous studies have focused primarily on radiological classification systems, biomechanical consequences of transitional anatomy, or surgical risk related to incorrect segment identification [22,31]. Functional correlation between imaging-defined nerve root compression and electrophysiologically identified radiculopathy has been largely overlooked [18,34]. This gap is clinically important because surgical decision-making often depends on accurate identification of the clinically relevant nerve root level. Failure to recognize anatomical–functional discordance may contribute to diagnostic uncertainty during surgical level determination. Although the present cohort was relatively small, it represents a highly selected group of patients who met strict diagnostic criteria, including whole-spine MRI-based vertebral numbering, clearly defined single-level nerve root compression on MRI, and single-root radiculopathy identified by EMG. These criteria were necessary to allow a precise level-to-level comparison between anatomical and functional findings and to minimize confounding from multilevel pathology or uncertain vertebral numbering. Therefore, the present findings should be interpreted as exploratory and hypothesis-generating rather than definitive, but they provide preliminary observations warranting further investigation in larger cohorts.

In this context, the present study provides clinical evidence suggesting that functional radiculopathy patterns frequently do not align with anatomically presumed levels in patients with LSTV. In the lumbarization group, S1 root compression was most frequently identified on MRI, whereas L5 radiculopathy was the predominant EMG finding. Similarly, in the sacralization group, patients with L4 root compression frequently demonstrated L5 radiculopathy on EMG. These findings support the concept that morphological resemblance may be more clinically relevant than conventional vertebral numbering alone. In other words, the functional identity of a nerve root may be better understood according to transitional morphology rather than strict anatomical labelling. By systematically evaluating the relationship between MRI findings and EMG results, this study highlights the limitations of relying exclusively on anatomical imaging in patients with transitional anatomy and extends the conceptual framework of LSTV beyond purely morphological classification [15,16].

From a clinical standpoint, the findings of this study may have potential relevance during diagnostic evaluation in selected patients. Reliance solely on anatomical imaging to determine the symptomatic level in patients with transitional vertebrae may complicate localization of the clinically relevant symptomatic level [35,36]. In selected cases, electrodiagnostic evaluation may provide valuable complementary information by reflecting the functional status of nerve roots and clarifying discrepancies between morphological findings and clinical presentation [37,38]. Integration of anatomical and functional assessments may provide complementary information during diagnostic evaluation, particularly in patients with complete or complex transitional morphology where anatomical–functional discordance is more likely to occur. In particular, patients with complete transitional morphology may require more careful preoperative evaluation, as the likelihood of anatomical–functional discordance appears to be substantially higher in this group. When MRI findings do not fully explain the patient’s symptoms or neurological deficits, additional electrophysiological assessment may assist in interpreting discrepancies between imaging findings and clinical presentation. This may be relevant in selected patients in whom imaging findings do not adequately explain clinical symptoms. A diagnostic strategy based solely on conventional vertebral numbering may not fully account for potential anatomical –functional discordance in patients with LSTV, whereas integrated interpretation of MRI, EMG, and clinical findings may support more comprehensive clinical evaluation. Rather than using EMG as a definitive diagnostic standard, it may be more appropriate to consider it as an adjunctive tool that helps interpret clinically relevant discrepancies between MRI findings and patient symptoms. Such an integrated diagnostic strategy may support more individualized diagnostic reasoning. However, whether this approach improves surgical outcomes or reduces wrong-level surgery requires prospective validation. Ultimately, recognition of the potential for discordance between imaging and electrophysiological findings may contribute to more comprehensive diagnostic evaluation in selected patients with LSTV, rather than relying solely on conventional anatomical assumptions [39].

Several limitations of the present study should be acknowledged. First, the retrospective single-centre design introduces potential selection bias and limits the generalizability of the findings. Because all data were derived from a single institution, the results may reflect institution-specific diagnostic practices, referral patterns, and patient characteristics rather than universally applicable clinical patterns. Therefore, the findings should be interpreted with caution when applied to other clinical settings. Second, the relatively small sample size may reduce statistical power and limit the robustness of subgroup analyses. This limited cohort was largely attributable to the strict inclusion criteria applied in this study, including whole-spine MRI-based vertebral numbering, clearly defined single-level nerve root compression on MRI, and single-root radiculopathy identified by EMG. Although these criteria were necessary to enable accurate level-to-level comparison between anatomical and functional findings, they inevitably reduced the number of eligible patients. This limitation may also have reduced the ability to detect potential associations with other anatomical variables, such as conus medullaris level, iliac crest level, or number of ribs. Although post hoc power analysis was performed for the primary subgroup comparison, the overall sample size remained limited, and the findings should therefore be interpreted cautiously. Therefore, the findings should be interpreted as hypothesis-generating observations rather than definitive prevalence estimates. Third, EMG is inherently operator-dependent and may be influenced by factors such as timing of evaluation, chronicity of pathology, and muscle sampling strategies. In addition, not all nerve root pathologies are detectable by EMG. EMG-detected radiculopathy may preferentially reflect functionally significant or more advanced cases, and subclinical or purely sensory involvement may not be identified. Therefore, some cases of radiculopathy may have been missed in this study. Fourth, the identification and classification of lumbosacral transitional vertebrae on MRI may vary depending on vertebral numbering methodology, particularly in cases with borderline or ambiguous morphology. In addition, formal inter- and intra-rater reliability analyses for MRI interpretation were not performed because of the retrospective design of the study, which may have influenced the consistency of imaging-based level determination. Fifth, clinical outcome data were not available in this study; therefore, it remains unclear whether identification of MRI–EMG discordance directly influences treatment decisions or improves postoperative outcomes. Although the present study suggests that integrated anatomical and functional evaluation may improve diagnostic reasoning, prospective studies are required to determine whether this approach reduces wrong-level surgery or improves postoperative functional recovery. In addition, because this study was based on cross-sectional radiological and electrophysiological assessments, it could not fully capture the dynamic relationship between structural compression and functional nerve root involvement over time. Future prospective multicenter studies with larger cohorts and standardized vertebral numbering protocols are required to validate these findings and to further elucidate the biomechanical and neurophysiological mechanisms underlying potential functional variation in nerve roots in transitional anatomy. Further prospective validation is necessary before these findings can be translated into routine clinical decision-making.

## 5. Conclusions

In this exploratory cohort, patients with lumbosacral transitional vertebrae showed a high rate of discordance between MRI-identified nerve root compression and EMG-identified radiculopathy. The degree of transitional morphology appears to influence this relationship, with complete transitions showing greater functional–anatomical discordance. These findings suggest that transitional vertebral anatomy may contribute to altered functional expression of radiculopathy and should be recognized during clinical interpretation of imaging and electrophysiological findings. Integration of anatomical imaging with functional assessment may provide complementary information in selected patients with LSTV.

## Figures and Tables

**Table 1:** Baseline clinical, demographic, and radiologic characteristics of the study population.

Variables	Category	Value
Age (Years)	Mean ± SD	57.9 ± 12.6
Sex	Female:Male	5:24
Underlying pathology	Disc herniation	24
	Lateral recess stenosis	3
	Foraminal stenosis	2
Level of nerve root compression on MRI	L4	5
	L5	8
	S1	13
	S2	3
EMG-identified radiculopathy level	L4	1
	L5	22
	S1	6
Presenting symptom	Pain only	18
	Pain with ADF weakness	1
	Pain with GTE weakness	1
	Pain with ADF, GTE weakness	8
	Pain with APF weakness	1
LSTV type	Lumbarization	19
	Sacralization	10
Number of ribs	11	4
	12	25
Iliac crest level	L3–4 disc	1
	L4 vertebral body	7
	L4–5 disc	6
	L5 vertebral body	11
	L5–S1 disc	3
	S1 vertebral body	1
Conus medullaris level	T12 vertebral body	3
	T12–L1 disc	2
	L1 vertebral body	7
	L1–2 disc	6
	L2 vertebral body	7
	L2–3 disc	4

Data are presented as the mean ± standard deviation or as the number of patients. SD, standard deviation; MRI, magnetic resonance imaging; EMG, electromyography; ADF, ankle dorsiflexion; GTE, great toe extension; APF, ankle plantarflexion; LSTV, lumbosacral transitional vertebrae.

**Table 2:** Distribution of EMG radiculopathy and dominant muscle involvement according to the type of LSTV.

Type of LSTV	EMG-Identified Radiculopathy Level	Dominant Muscles Involved
Lumbarization (*n* = 19)	L4 (*n* = 1)	PL (1), TA (1), VL (1)
	L5 (*n* = 13)	PL (12), TA (11), TFL (5), EHL (3)
	S1 (*n* = 5)	GC (5), GM (4), PL (4)
Sacralization (*n* = 10)	L5 (*n* = 9)	PL (9), TA (8), TFL (8)
	S1 (*n* = 1)	GC (1), GM (1), FDL (1)

Data are presented as the number of patients. Muscle counts indicate the number of patients in whom the corresponding muscle showed electrophysiological involvement. EMG, electromyography; LSTV, lumbosacral transitional vertebrae; PL, peroneus longus; TA, tibialis anterior; VL, vastus lateralis; TFL, tensor fasciae latae; EHL, extensor hallucis longus; GC, gastrocnemius; GM, gluteus maximus; FDL, flexor digitorum longus.

**Table 3:** Comparison of Variables Associated with Concordance between MRI and EMG Findings (*n* = 29).

Variables	Concordant (*n* = 8)	Discordant (*n* = 21)	*p*-Value
Age (Years)	55.1 ± 10.5	58.6 ± 13.1	0.502
Sex (Female:Male)	3:5	6:15	0.642
Motor weakness (Absent:Present)	5:3	13:8	1.000
Type of LSTV (Lumbarization:Sacralization)	4:4	15:6	0.278
Degree of LSTV (Complete:Partial)	1:7	15:6	0.010 *
Conus medullaris level (High:Normal:Low)	5:2:1	13:4:4	0.885
Iliac crest level (High:Normal:Low)	4:0:4	11:4:6	0.316
Number of ribs (11:12)	2:6	2:19	0.300

Data are presented as the mean ± standard deviation or as the number of patients. * indicates statistical significance. MRI, magnetic resonance imaging; EMG, electromyography; LSTV, lumbosacral transitional vertebrae.

## Data Availability

The data underlying this article cannot be shared publicly because of the privacy of the individuals who participated in this study. The data can be shared by the corresponding authors upon reasonable request.
